# First‐line sorafenib sequential therapy and liver disease etiology for unresectable hepatocellular carcinoma using inverse probability weighting: A multicenter retrospective study

**DOI:** 10.1002/cam4.4367

**Published:** 2021-10-24

**Authors:** Shigeo Shimose, Atsushi Hiraoka, Masahito Nakano, Hideki Iwamoto, Masatoshi Tanaka, Takaaki Tanaka, Kazunori Noguchi, Hajime Aino, Kei Ogata, Masahiko Kajiwara, Satoshi Itano, Yoshinori Yokokura, Taizo Yamaguchi, Hiroshi Kawano, Norito Matsukuma, Hideya Suga, Takashi Niizeki, Tomotake Shirono, Yu Noda, Naoki Kamachi, Shusuke Okamura, Takumi Kawaguchi, Hironori Koga, Takuji Torimura

**Affiliations:** ^1^ Division of Gastroenterology Department of Medicine Kurume University School of Medicine Kurume Fukuoka Japan; ^2^ Gastroenterology Center Ehime Prefectural Central Hospital Matsuyama Japan; ^3^ Iwamoto Internal Medical Clinic Kitakyusyu Japan; ^4^ Clinical Research Center Yokokura Hospital Miyama Fukuoka Japan; ^5^ Department of Gastroenterology Omuta City Hospital Omuta Japan; ^6^ Division of Gastroenterology Department of Medicine Social Insurance Tagawa Hospital Tagawa Japan; ^7^ Department of Gastroenterology Kurume University Medical Center Kurume Japan; ^8^ Department of Gastroenterology Chikugo City Hospital Chikugo Japan; ^9^ Department of Gastroenterology Kurume Central Hospital Kurume Japan; ^10^ Department of Gastroenterology St. Mary's Hospital Kurume Japan; ^11^ Department of Gastroenterology Kurume General Hospital Kurume Japan; ^12^ Department of Gastroenterology and Hepatology Yanagawa Hospital Yanagawa Japan

**Keywords:** carcinoma, hepatocellular, nonalcoholic steatohepatitis, sequential therapy, sorafenib

## Abstract

**Background and Aims:**

Sequential therapy with molecular‐targeted agents (MTAs) is considered effective for unresectable hepatocellular carcinoma (HCC) patients. This study purposed to evaluate the efficacy of sequential therapy with sorafenib (SORA) as a first‐line therapy and to investigate the therapeutic impact of SORA in nonalcoholic fatty liver disease (NAFLD) or nonalcoholic steato hepatitis (NASH)‐related HCC.

**Methods:**

We evaluated 504 HCC patients treated with SORA (Study‐1). The times of administration for sorafenib from 2009 to 2015, 2016 to 2017, and 2018 and later were defined as the early‐, mid‐, and late‐term periods, respectively. Among them, 180 HCC patients treated with SORA in addition to MTAs in the mid‐ and late‐term periods were divided into groups based on disease etiology (NAFLD or NASH [*n* = 37] and viral or alcohol [*n* = 143]), and outcomes were compared after inverse probability weighting (IPW) (Study‐2).

**Results:**

Overall survival (OS) of HCC patients who received sequential MTA therapy after first‐line SORA was significantly longer. The median survival times (MST) were 12.6 versus 17.6 versus 17.4 months in the early‐term group, mid‐term group, and the later‐time group (early vs. mid, *p* = 0.014, early vs. later. *p* = 0.045), respectively. (Study‐1). In Study‐2, there was no significant differences in OS between the Virus/alcohol group and the NAFLD/NASH group in patients who received sequential therapy (MST was 23.4 and 27.0 months *p* = 0.173, respectively). The NAFLD or NASH, female sex, albumin‐bilirubin (ALBI) grade 2b, and major Vp (Vp3/Vp4) were significant factors for OS treated with SORA.

**Conclusions:**

Sequential therapy with SORA as the first‐line treatment improved the prognosis of unresectable HCC patients and was effective regardless of HCC etiology.

## Graphical abstract

Sequential therapy with SORA as the first‐line treatment prolonged the prognosis of unresectable HCC patients and was effective regardless of HCC etiology.

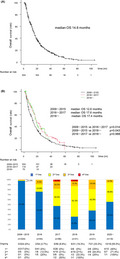

## 1 INTRODUCTION

As the incidence of hepatocellular carcinoma (HCC) has increased, HCC has been the most common malignant tumor worldwide.^1^, ^2^ Although the prognosis of unresectable HCC patients remains unsatisfactory,^3^ recently, various molecular‐targeted agents (MTAs) have been approved for this condition. Sorafenib (SORA) was one of the first drugs to be approved as first‐line therapy for unresectable HCC patients^4^; Currently, five types of MTAs are available in Japan to treat patients with unresectable HCC. Moreover, in 2020, combination therapy with atezolizumab plus bevacizumab was shown to significantly prolong progression‐free survival (PFS) and overall survival (OS) compared to SORA.^5^

The combination immunotherapy with atezolizumab and bevacizumab, which act as immune checkpoint inhibitors (ICIs), has also been approved for unresectable HCC patients as first‐line therapy, following results of the IMbrave 150 trial.^5^ However, new and interesting insights have recently shed light on the therapeutic response to immunotherapy for HCC. Dominik et al. reported that the response to immunotherapy differed depend on the liver etiology.^6^ Notably, nonalcoholic steatohepatitis (NASH)‐related HCC showed less response to immunotherapy, resulting in a shortened median survival time (MST).^6^ Based on these results, immunotherapy may not be the best first‐line treatment for NASH‐related HCC.

Sequential treatment with multiple MTAs can prolong median OS for patients with unresectable HCC.^7^, ^8^ SORA is the most well‐known and documented MTA used in sequential systemic treatment for HCC.^9^ Sequential therapy typically progresses from SORA to regorafenib (REGO), lenvatinib (LEN), and finally transcatheter arterial chemoembolization. However, the IMbrave 150 trial showed that atezolizumab plus bevacizumab was superior to SORA for OS. Interestingly, this combination did not show superiority to SORA in patients with a nonviral disease etiology.^10^ These results suggest that the HCC‐related etiology is a significant element in the proper administration of MTA and ICIs. However, it is still unknown whether the therapeutic effects of SORA in HCC may differ according to liver etiology, especially NAFLD or NASH‐related HCC patients.

In this study, we aimed to investigate the benefits of sequential MTA therapy, following the use of SORA as first‐line therapy. Additionally, we evaluated differences in the therapeutic effects of SORA as a first‐line sequential therapy based on the etiology of viral and nonviral liver disease, especially NAFLD or NASH, using inverse probability weighting (IPW).

## 2 METHODS

### 2.1 Study design and patients

Between 2009 and 30 October 2020, this study retrospectively included 714 HCC patients who received SORA as first‐line therapy, at 12 independent Japanese institutions: Kurume University Hospital, Ehime Prefectural Central Hospital, Omuta City Hospital, Iwamoto Internal Medical Clinic, Yokokura Hospital, Social Insurance Tagawa Hospital, Kurume University Medical Center, Chikugo City Hospital, Kurume Central Hospital, Kurume General Hospital, St. Yanagawa Hospital, and Mary's Hospital. The following criteria were excluded: Child‐Pugh class B or C (*n* = 129), intolerance to SORA (*n* = 54), autoimmune hepatitis or primary biliary cirrhosis (*n* = 4), or lost to follow‐up (*n* = 23). Thus, 504 enrolled patients were evaluated and analyzed (Study‐1). In addition, patients who were treated with SORA before 2015 were excluded from the analysis (Study‐2). The cutoff date of this study was 31 March 2021.

**FIGURE 1:**
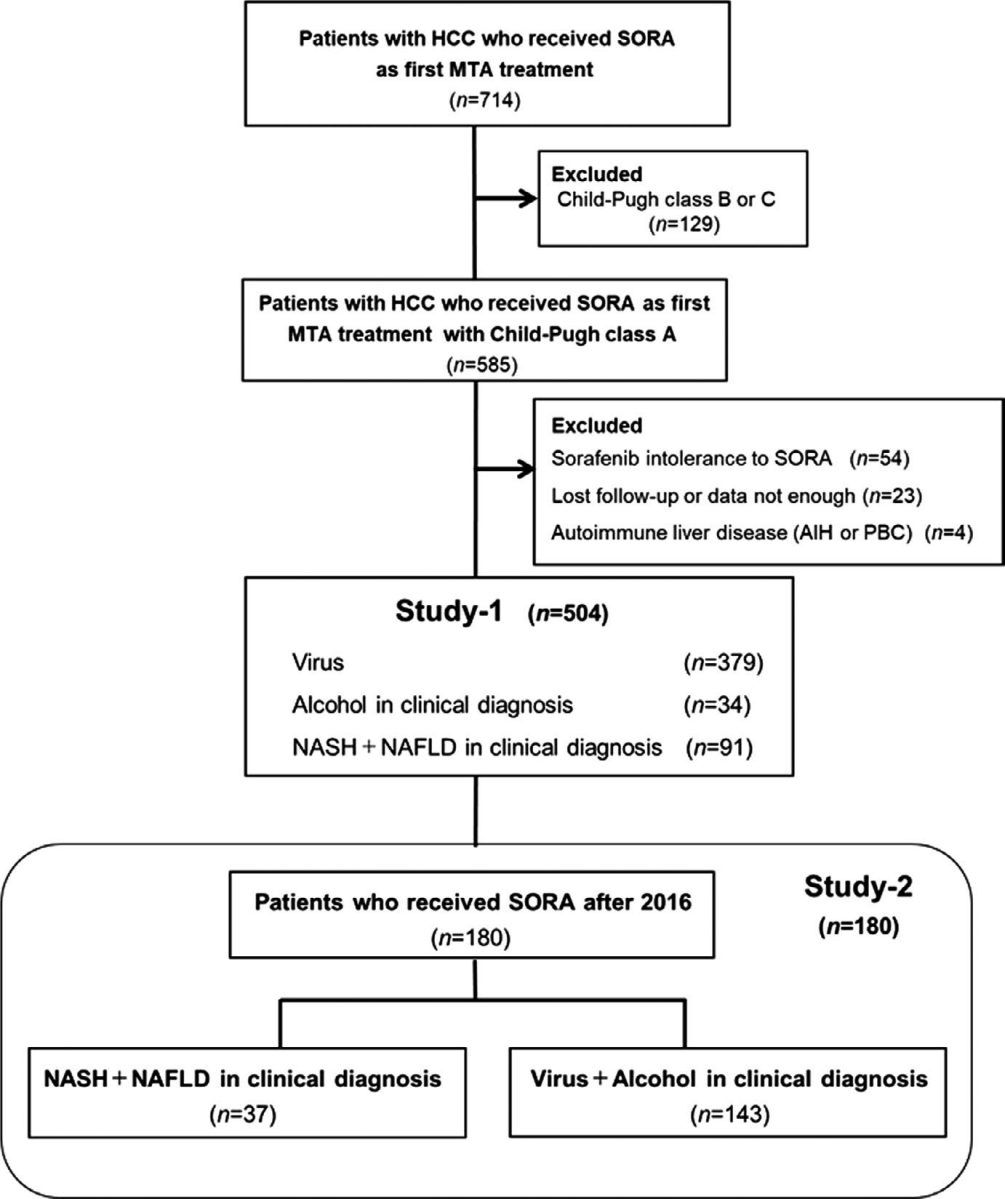
Study design. A total of 714 patients with HCC were evaluated and treated with SORA as the first‐line MTA treatment. In the course of the study, 210 patients were excluded, and 504 patients with HCC were enrolled in Study‐1. Study‐2 included 180 patients who received SORA after 2016. Abbreviations: MTA, molecular‐targeted agent; SORA, sorafenib

To determine whether disease etiology affected the response to SORA, 180 HCC patients treated with SORA as first‐line therapy were enrolled in Study‐2. Cases were allocated to a group with a clinical diagnosis of NAFLD or NASH disease etiology (*n* = 37) or a group with a clinical diagnosis of Virus, or Alcohol disease etiology (*n* = 143) (Figure 1). The study was conducted following the Helsinki Declaration and approval of the Ethical Committee of Kurume University School of Medicine (approval code: 21074). We obtained informed consent using an opt‐out approach.

### 2.2 Assessment of liver disease etiology

Alcoholic liver disease was diagnosed for patients with an alcohol intake history of 60 g/day or more.^11^ For a diagnosis of NAFLD/NASH, this comprehensive analysis was used: (1) a medical interview on lifestyle‐related diseases^12^ (history of obesity, diabetes mellitus, hypertension, hyperlipidemia, etc.), (2) low history of alcohol (<20 g/day and <30 g/day in women and men, respectively), (3) fatty liver diagnosed by ultrasonography, and (4) NAFLD or NASH diagnosed according to pathological findings.^13^

### 2.3 Assessment of hepatic functional reserve

The hepatic functional reserve was evaluated using the albumin‐bilirubin (ALBI) score. The modified ALBI grade was defined used ALBI score as following; ALBI grade 1 = ≤−2.60, ALBI grade 2a = >−2.60 to ≤−2.27, ALBI grade 2b = >−2.27 to ≤−1.39, ALBI grade 3 = >−1.39.^14^

### 2.4 Definition of the chronological order of administration of SORA

The times of administration of SORA from 2009 to 2015, 2016 to 2017, and 2018 later were defined as the early‐, mid‐, and late‐term periods, respectively.

### 2.5 Treatment protocol

SORA was orally administered to patients according to the manufacturer's instructions. Adverse events (AEs) were evaluated using the National Cancer Institute Common Terminology Criteria for Adverse Events (version 5.0), and dose reduction or temporary interruption was implemented when any AEs were observed.

### 2.6 Efficacy of SORA and observation schedule

The therapeutic response of HCC was evaluated according to the modified Response Evaluation Criteria in Solid Tumors^15^ using CT or MRI 4–6 weeks after the initial treatment. After that, evaluation occurred every 3 months until death (31 March 2021).

### 2.7 Statistical analysis

Statistical analyses were conducted by Easy R (EZR), (EZR, version 1.53), A graphical user interface for R (The R Foundation for Statistical Computing).^16^ Fischer's exact test, Welch's *t*‐test, Student's *t*‐test, and Mann–Whitney's *U*‐test were used, and Bonferroni's test was used for multiple comparisons groups. Survival times were calculated using the Kaplan–Meier method, the log‐rank test, and Cox hazard analysis (stepwise regression method). Logistic regression analyses including ALBI score, age, sex, and Barcelona Clinic Liver cancer (BCLC) stage were conducted to calculate probabilities for the NAFLD/NASH and Virus/Alcohol groups. IPW was defined as 1/(propensity score) for the NAFLD/NASH group and 1/(1‐propensity score) for the Virus/Alcohol group. A two‐tailed *p*‐value of <0.05 was considered statistically significant. OS was determined depended on IPW‐adjusted analysis.^17^

## 3 RESULTS

### 3.1 Study‐1

#### 3.1.1 Patients

Baseline characteristics of 504 patients are summarized in Table 1. The median age was 72 years. The etiology of HCC was hepatitis B virus (HBV), hepatitis C virus (HCV), HBV + HCV, alcohol, and NAFLD or NASH in 88 patients (17.4%), 288 patients (57.1%), 3 patients (0.6%), 34 patients (6.8%), and 91 patients (18.1%), respectively. The ALBI grade was 1 in 191 patients (37.9%), 2a in 162 patients (32.2%), and 2b in 151 patients (29.9%). BCLC stage C was observed in 63.0% (316/504) of the patients. Macrovascular invasion (MVI) and extrahepatic spread (EHS) were present in 93 patients (18.4%) and 265 patients (52.5%), respectively (Table 1).

**TABLE 1:** Patient characteristics

Characteristic	All patients	Virus/Alcohol	NAFLD/NASH	*p*
*N*	504	413	91	
Age (years old)	72 (33–91)	71 (33–91)	74 (45–87)	0.030
Sex (female/male)	88/416	72/341	16/75	0.973
Etiology (HBV/HCV/HBV + HCV/Alcohol/NAFLD or NASH)	88/288/3/34/91			
ALBI score (median [range])	−2.48 (−3.61 to −1.47)	−2.47 (−3.61 to −1.47)	−2.50 (−3.19 to −1.68)	0.211
ALBI grade (1/2a/2b)	191/162/151	155/129/129	36/33/22	0.375
Tumor size (mm)	30 (10–190)	30 (10–190)	31 (10–180)	0.471
BCLC stage (A/B/C)	(6/180/318)	(4/145/264)	(2/35/54)	0.526
Macrovascular invasion (None/Vp1/Vp2/Vp3/Vp4)	(411/4/36/36/17)	(335/4/31/29/14)	(76/0/5/7/3)	0.709
Extrahepatic spread (Yes/No)	(265/239)	(216/197)	(49/42)	0.789
AFP (ng/ml)	90.4 (0.9–955,258)	84.4 (1.7–955,258)	133.9 (0.9–470,335)	0.081
DCP (mAU/ml)	432.5 (2–1590,000)	432 (2–1590,000)	443 (11–157,00)	0.351
Initial dose (200/400/600/800 mg)	18/337/15/134	14/278/14/107	4/59/1/27	0.574
Pretreatment (Yes/No)	(481/23)	(394/19)	(87/4)	0.932

Note

Data are expressed as median (range) or number.

Abbreviations: AFP, α‐fetoprotein; ALBI, Albumin‐bilirubin; Albumin‐bilirubin score; BCLC, Barcelona Clinic Liver Cancer; DCP, des‐γ‐carboxy prothrombin; HBV, hepatitis B virus; HCV, hepatis C virus; NAFLD, nonalcoholic fatty liver disease; NASH, nonalcoholic steatohepatitis.

The Virus or Alcohol and the NAFLD or NASH groups included 403 and 91 patients, respectively. The median age was only significantly different between the virus or alcohol group and the NAFLD or NASH group (Table 1).

#### 3.1.2 Evaluation of the therapeutic response to SORA according to liver disease etiology

Therapeutic responses to SORA are shown in Figure S1. There were no significant differences between the two groups in the overall objective response rate (ORR) and disease control rate (DCR) associated with liver disease etiology of either NAFLD/NASH or Virus/Alcohol (ORR: 5.6% vs. 8.7%, *p* = 0.287; DCR: 44.0% vs. 47.4%; *p* = 0.572, Figure S1).

#### 3.1.3 Progression‐free survival and treatment duration with SORA

PFS was 3.5 months (Figure S2A). The median duration of SORA was 4.5 months.

#### 3.1.4 Overall survival with SORA according to the time period of treatment

**FIGURE 2:**
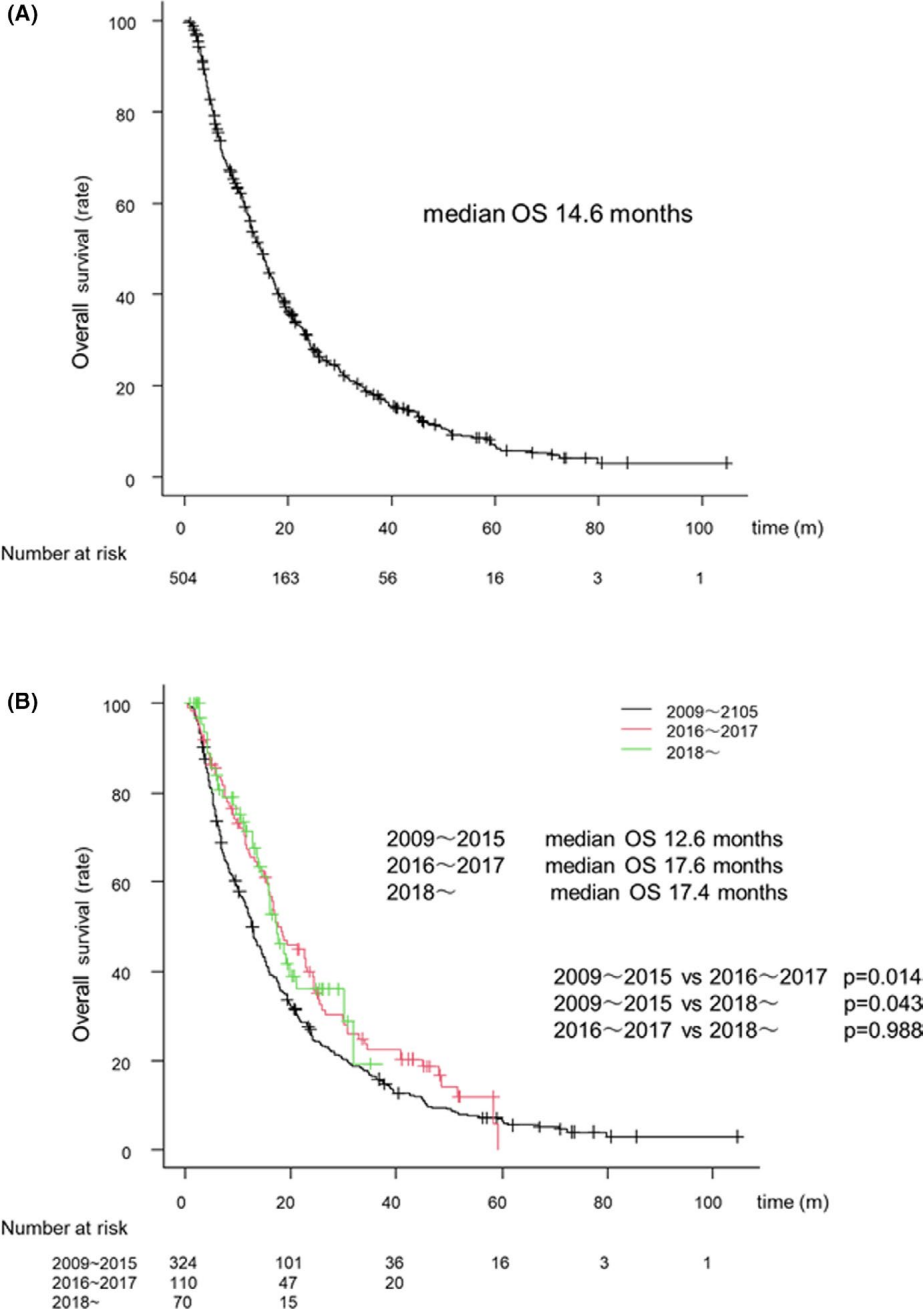
(A) Overall survival (OS) of HCC patients treated with SORA. (B) Survival in HCC patients treated with SORA in the different periods. OS for the 2009 to 2015 group, the 2016 to 2017 group, and the 2018 later group. The black, red, and green lines indicate the 2009 to 2015 group, the 2016 to 2017 group, and the 2018 and later group, respectively. Abbreviation: SORA, sorafenib

The MST of all patients was 14.6 months after treatment with SORA (Figure 2A). The MST was 12.6, 17.6, and 17.4 months in the 2009–2015, 2016–2017, and 2018 groups, respectively (Figure 2B).

#### 3.1.5 Conversion rate to sequential MTA therapy

**FIGURE 3:**
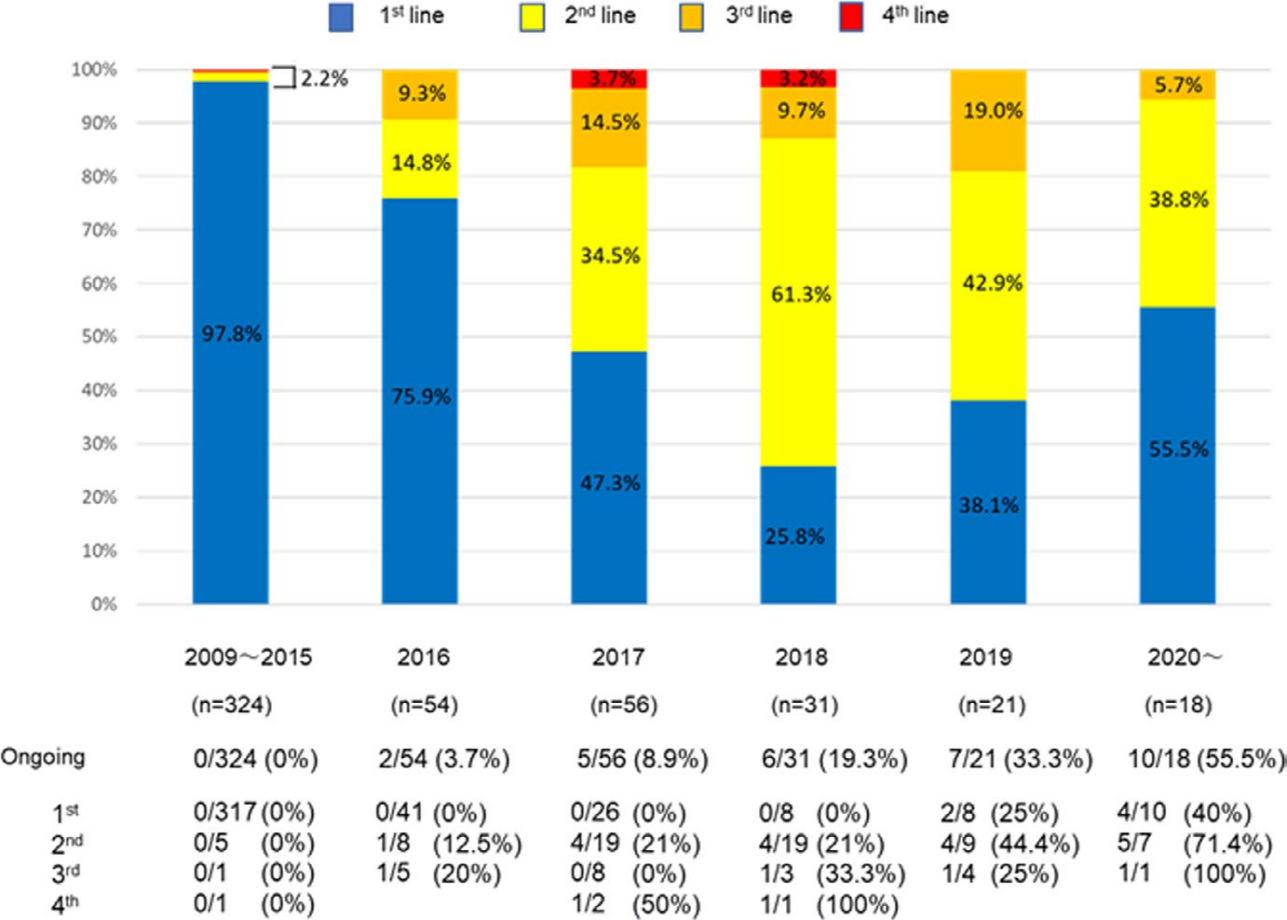
Conversion rates for sequential MTA therapy. The blue, yellow, orange, and red blocks indicate the 1st, 2nd, 3rd, and 4th groups, respectively. Abbreviation: MTA, molecular‐targeted agent

The conversion rate to sequential MTA therapy is shown in Figure 3. Sequential therapy could not be administered to most patients who had received SORA prior to 2015 because second‐line MTAs were not yet available. The percentage of patients that received sequential therapy was 24.1%, 52.7%, and 74.2% in 2016, 2017, and 2018, respectively. Sequential MTA therapy was ongoing for 33.3% and 55.6% of patients in 2019 and 2020, respectively, until the study censor time (Figure 3).

### 3.2 Study‐2

#### 3.2.1 Patients

The background of the 180 patients in study‐2 is summarized (Table 2). The Virus or Alcohol and the NAFLD or NASH groups had 143 and 37 patients, respectively, and 47.7% of patients (86/180) received sequential therapy after SORA. The median age differed significantly between the groups. However, another background of patients’ characteristics were similar between the two groups (Table 3).

**TABLE 2:** Patient characteristics

Characteristic	All patients	Virus/Alcohol	NAFLD/NASH	*p*
*n*	180	143	37	
Age (years old)	72 (36–91)	71 (36–91)	76 (61–87)	0.006
Sex (female/male)	31/149	27/116	4/33	0.246
Etiology (Virus/Alcohol/NAFLD + NASH)	124/19/37	124/19/0	0/0/37	<0.001
ALBI score (Median [range])	−2.54 (−3.61 to −1.47)	−2.55 (−3.61 to −1.47)	−2.48 (−3.19 to −1.68)	0.963
ALBI grade (1/2a/2b)	80/50/50	64/39/40	16/11/10	0.957
Tumor size (mm)	31 (10–190)	32 (10–190)	27.5 (10–150)	0.445
BCLC stage (A/B/C)	3/80/97	2/62/79	1/18/18	0.696
Macrovascular invasion (None/major Vp(−)/major Vp(+))	147/16/17	116/14/13	31/2/4	0.566
Extrahepatic spread (Yes/No)	79/101	63/80	16/21	0.929
AFP (ng/ml)	77.2 (0.9–470,335)	101.4 (0.9–177,630)	27.7 (0.9–470,335)	0.208
DCP (mAU/ml)	278 (3.9–89,928)	278 (3.9–54,583)	285 (13–89,928)	0.762
IPW		4.04 (3.37–6.05)	1.22(1.13–1.35)	<0.001

Note

Data are expressed as median (range), or number.

Abbreviations: AFP, α‐fetoprotein; ALBI, Albumin‐bilirubin; Albumin‐bilirubin score; BCLC, Barcelona Clinic Liver Cancer; DCP, des‐γ‐carboxy prothrombin; IPW, inverse probability weighting; NAFLD, nonalcoholic fatty liver disease; NASH, nonalcoholic steatohepatitis.

**TABLE 3:** Cox hazard analysis for OS

Factors	HR	95% Confidence interval	*p*
Age ≥ 75 years	1.62	1.04–2.52	0.033
Female gender	0.72	0.39–1.13	0.303
m‐ALBI grade 2	1.89	1.20–2.98	0.005
BCLC stage C	0.82	0.45–1.51	0.529
AFP ≥ 400 ng/ml	2.47	1.55–3.94	0.001
DCP ≥ 400 ng/ml	1.14	0.73–1.76	0.565
Major Vp (Vp3/Vp4)	2.49	1.38–4.48	0.002
Extrahepatic spread	1.25	0.79–1.98	0.337
NAFLD/NASH	0.66	0.39–1.14	0.138
Results of stepwise regression method			
NAFLD/NASH	0.48	0.29–0.81	0.005
Female	0.59	0.29–0.99	0.048
ALBI grade 2b	2.12	1.42–3.17	<0.001
Major Vp (Vp3/Vp4)	2.79	1.53–5.08	<0.001

Abbreviations: AFP, α‐fetoprotein; BCLC, Barcelona Clinic Liver Cancer; DCP, des‐γ‐carboxy prothrombin; m‐ALBI, modified Albumin‐bilirubin; NAFLD, nonalcoholic fatty liver disease; NASH, nonalcoholic steatohepatitis; OS, overall survival.

#### 3.2.2 Progression‐free survival and treatment duration with SORA

The median PFS was 3.8 months (Figure S2B). The median duration time of SORA treatment was 4.9 months.

#### 3.2.3 Overall survival with SORA by liver disease etiology before IPW

**FIGURE 4:**
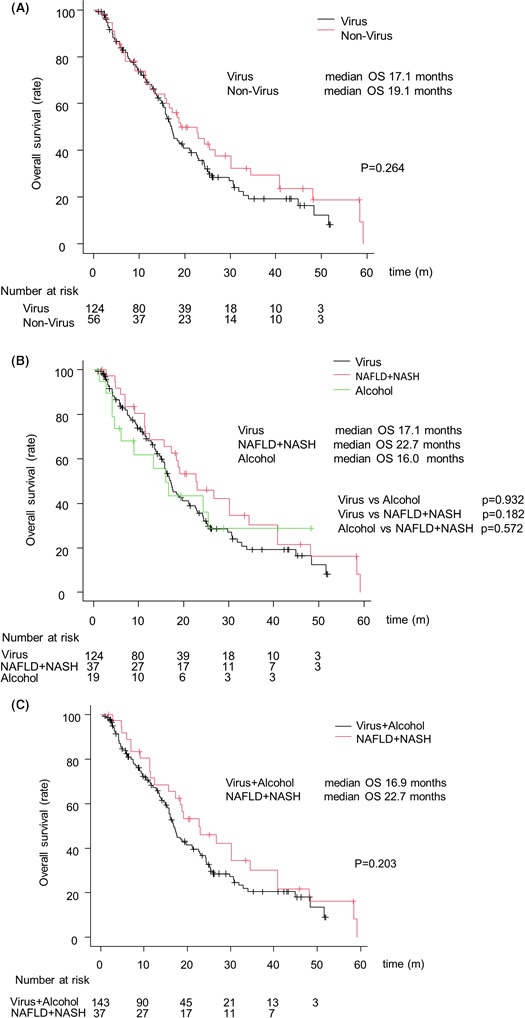
Overall survival (OS) of HCC patients treated with SORA after 2016. (A) OS according to the virus group and non‐virus group. The black and red lines indicate the virus group and non‐virus, respectively. (B) OS according to the virus, alcohol, and the NAFLD/NASH groups. The black, green, and red lines indicate the virus, alcohol, and the NAFLD + NASH groups, respectively. (C) OS according to the virus/alcohol and NAFLD/NASH groups. The black and red lines indicate the virus/alcohol and NAFLD/NASH groups, respectively. Abbreviation: SORA, sorafenib

OS and association with SORA treatment after 2016 are shown in Figure 4A–C. There were no significant differences between the virus and non‐virus groups (MST: 17.1 vs. 19.1 months, *p* = 0.264, respectively; Figure 4A). Additionally, OS between the virus groups and the alcohol and NAFLD/ NASH groups were similar (MST: 17.1, 16.0, and 22.7 months, respectively; Figure 4B). Since no significant difference was shown in OS between the virus and the alcohol group detected using the Bonferroni methods, and the virus and the alcohol group were also similar, these groups were combined. Based on these results also showed no significant difference between the Virus or Alcohol and the NAFLD or NASH groups (MST: 16.9 and 22.7 months, *p* = 0.203, respectively; Figure 4C).

#### 3.2.4 Overall survival with SORA according to liver disease etiology after IPW

**FIGURE 5:**
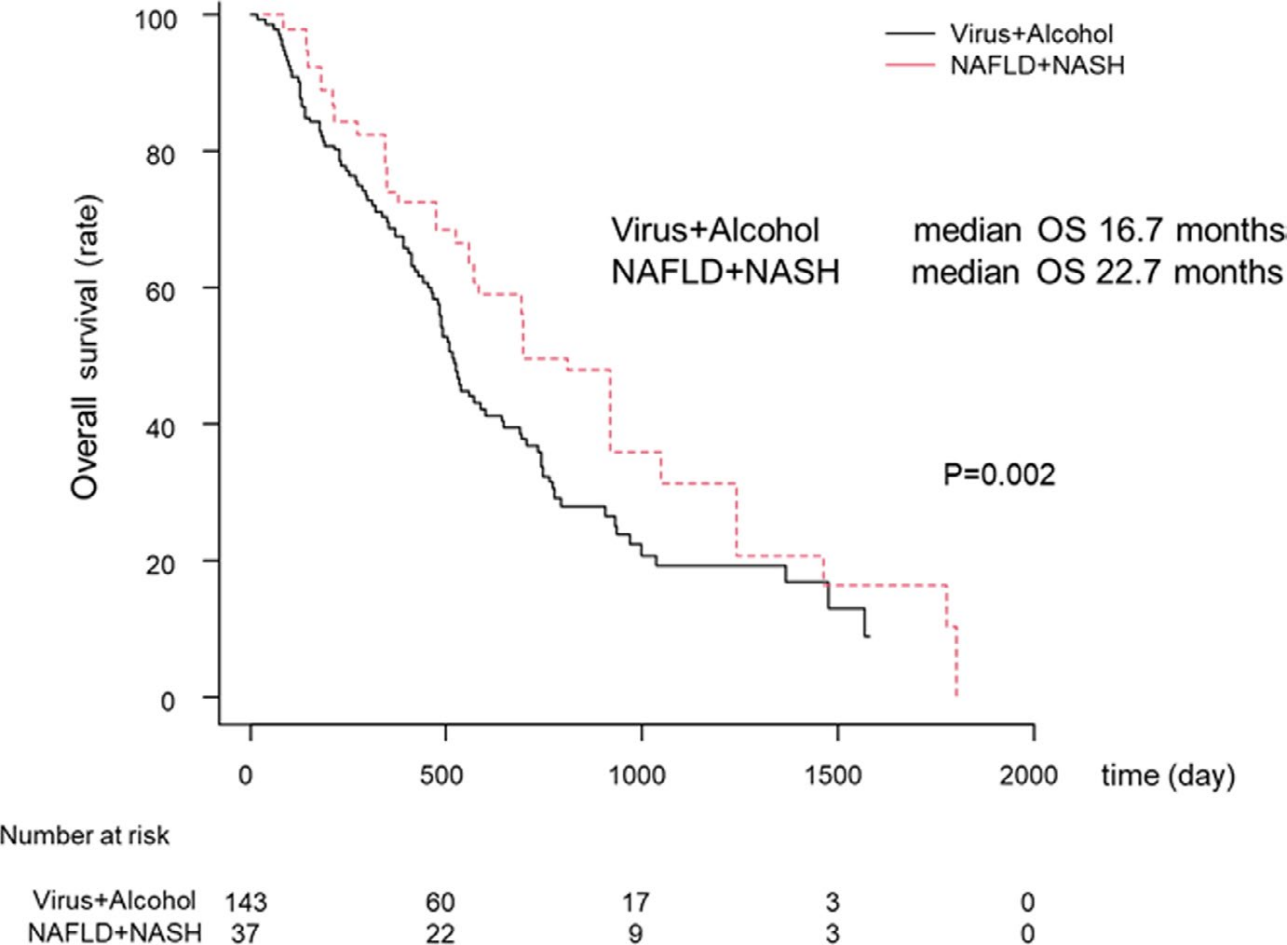
Overall survival (OS) of HCC patients treated with SORA after 2016 after IPW. OS according to the virus/alcohol and NAFLD/NASH groups. The black and red lines indicate the virus/alcohol and NAFLD/NASH groups, respectively. Abbreviation: SORA, sorafenib

*OS* was significantly longer in the NAFLD/NASH group than in the Virus/Alcohol group (MST: 23.3 vs. 17.2 months, *p* = 0.005; Figure 5).

#### 3.2.5 Overall survival with SORA according to the Virus/Alcohol and the NAFLD/NASH groups in patients with sequential therapy.

**FIGURE 6:**
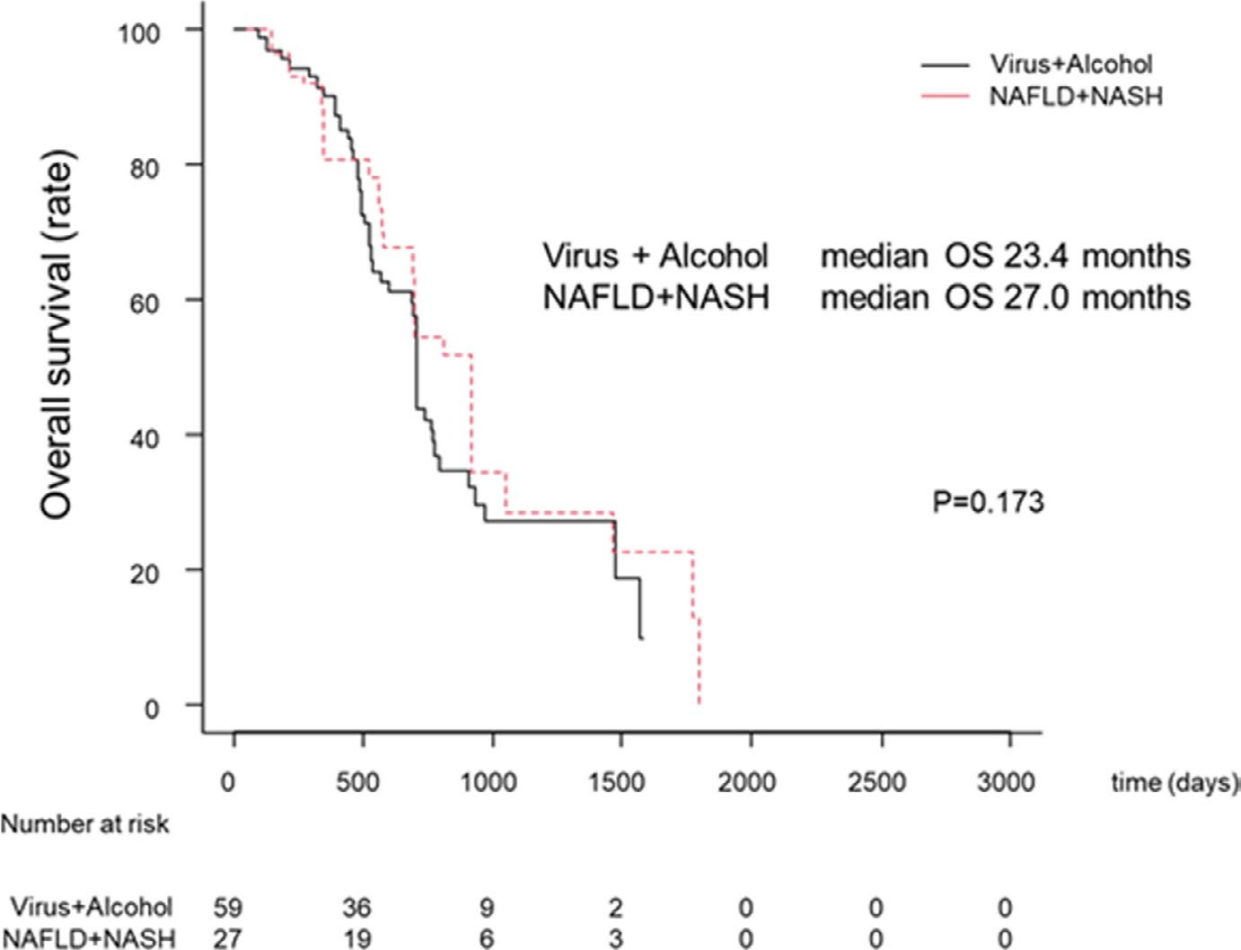
Overall survival (OS) according to the Virus/Alcohol and the NAFLD/NASH groups in patients with sequential therapy. The black and red lines indicate the virus/alcohol and the NAFLD/NASH groups, respectively

In patients who received sequential therapy, no significant differences in OS between the Virus/alcohol group and the NAFLD/NASH group after IPW (MST was 23.4 and 27.0 months *p* = 0.173, respectively, Figure 6).

#### 3.2.6 Cox regression analysis for survival after IPW

Cox regression analysis of OS was performed using the factors including age, sex, disease etiology, BCLC stage, m‐ALBI grade, AFP, DCP, major Vp, and EHS after IPW. The independent factors for OS are summarized in Table 3. NAFLD/NASH (HR 0.48; *p* = 0.005), female sex (HR 0.59; *p* = 0.048), ALBI grade 2b (HR 2.12; *p* < 0.001), and major Vp (Vp3/Vp4) (HR 2.79; *p* < 0.001) were identified using a stepwise procedure and logistic regression analysis (Table 3).

## 4 DISCUSSION

The present study found that sequential therapy with SORA as the first‐line treatment, followed by other MTAs, improved the prognosis of unresectable HCC patients. In addition, after IPW analysis, the NAFLD/NASH etiology, female sex, ALBI grade 2b, and major Vp were identified as significant factors for OS in HCC patients treated with SORA. Moreover, the study revealed that SORA treatment improved the prognosis of NAFLD or NASH‐related HCC patients.

Recently, with SORA as first‐line therapy, sequential therapy using MTAs has been considered effective for unresectable HCC patients.^8^, ^9^ In our study, most of the patients who were administered SORA prior to 2015 did not progress beyond SORA monotherapy. However, many patients who were administered SORA as first‐line therapy in 2016 and afterward could be administered second‐line and MTA therapies later. In 2018, 75.8% of the patients who received SORA as a first‐line therapy also received second‐line and later sequential therapy. In 2018, another first‐line therapy, LEN, was approved in Japan for HCC. Also, various second‐line and newer MTAs such as REGO, ramucirumab (RAM), and cabozantinib (CAB) have become available as standard systemic therapies following initial SORA or LEN treatment for unresectable HCC.^18^, ^19^, ^20^ Indeed, SORA first‐line therapy in the mid‐term period (from 2016 to 2017) and in the late period (after 2018) is associated with prolonged OS compared to the early period (before 2015). It is also noteworthy that many HCC patients treated with SORA in the late period are presently still undergoing sequential therapy. Therefore, it is expected that multi‐MTAs sequential therapy can contribute to further prolongation of survival for patients with unresectable HCC.

This study identified that ALBI grade 2b and major Vp were independent factors for poor prognosis. We have previously shown that ALBI grade is one of the significant indicators for HCC prognosis.^21^ Ogasawara et al. also reported that ALBI grade 2b was an independent predictor of poor prognosis in patients treated with SORA.^22^ In addition, several reports have described that MVI is a negative factor for OS in patients treated with SORA.^22^, ^23^ Nakazawa reported that the MST of HCC patients with major MVI was only 4.8 months.^24^ Moreover, Kaneko et al. reported that MTA monotherapy did not prolong the survival of HCC patients with ALBI grade 2b/3 and the major Vp groups.^25^ Thus, ALBI grade is needed to predict the prognosis of HCC patients treated with SORA. A multidisciplinary therapeutic strategy is needed to treat advanced HCC with major Vp.

The present study showed that SORA prolonged OS in NAFLD or NASH HCC patients than in HCC patients with different etiologies. Despite the remarkable outcomes seen with the use of atezolizumab plus bevacizumab in the IMbrave150 study, the combination did not show superiority in regards to the survival of patients with non‐virus‐related HCC when compared to the virus‐related HCC patients.^5^ In the present Study‐2, since no significant difference was shown in OS between the virus and the alcohol group detected using the Bonferroni methods (Figure 4B) as well as a similar report analyzed for LEN,^26^ we compared the prognosis of the NAFLD or NASH‐related HCC patient group against that of the combined virus and alcohol‐related HCC patient group. It is unclear why SORA has different effects depending on liver disease etiology. However, we previously reported that progression‐free survival after LEN treatment was better in the NAFLD/NASH than in the Viral/Alcohol group.^26^ In addition, some clinical trials for MTA showed that nonviral‐related HCC had a better therapeutic effect or improved prognosis than viral‐related HCC.^18^, ^27^ These results showed that anti‐VEGF antibody and molecular‐targeted agents are effective for nonviral‐related HCC. Although these previous reports did not enroll HCC patients with NAFLD/NASH, 85% of patients with nonviral HCC have a character for NAFLD, suggesting that NAFLD/NASH is the dominant etiology for non‐B non‐C HCC.^28^ Thus, this observation may provide evidence for choosing between ICI and SORA as a first‐line drug and systemic treatment for HCC. In the subgroup analysis, 47.7% of patients (86/180) received sequential therapy after SORA. Of these, 60.2% (52/86) and 32.5% (28/86) were treated with sequential therapy with LEN and REGO as second‐line, respectively. Moreover, in patients who received sequential therapy, no significant differences in survival time between the Virus/alcohol group and the NAFLD/NASH group after IPW (MST was 23.4 and 27.0 months *p* = 0.173, respectively, Figure 6). Therefore, sequential therapy with SORA as the first‐line therapy has the potential to be effective for patients irrespective of HCC etiology. However, given the small number of NAFLD/NASH patients and the fact that the majority of NAFLD/NASH patients were clinically diagnosed, further studies are needed to support these findings.

NAFLD is possibly predicted to be one of the most causes of HCC in the coming decades.^29^, ^30^, ^31^ NASH is characterized by severe hepatocyte injury and is an inflammatory consequence of NAFLD. This condition is strongly associated with metabolic syndrome.^30^ The prevalence of lifestyle‐related diseases is an increasing cause of the development and progression of NAFLD or NASH. However, proper diagnostic methods have not yet been established. Thus, it is desirable to create a screening system to properly diagnose NASH. Furthermore, it is also necessary to establish effective therapeutic strategies for NAFLD/NASH‐related HCC patients.

There were several limitations in this study. First, this was a retrospective study. Second, the number patients of the NAFLD/NASH group was small, and most patients were clinically diagnosed with NAFLD/NASH (Only two patients (5.4%) with performed liver biopsy in this study‐2). Therefore, knowledge regarding the etiology of liver disease was limited, particularly for the non‐NAFLD/NASH group. Third, accurate diagnosis of overlapping causes, such as those with viral plus NAFLD/NASH or viral plus alcohol, was limited. Fourth, to enhance the generalizability of this study, we did not evaluate Child‐Pugh class B (especially, B7) cases with relatively preserved hepatic function, which are considered to tolerate sorafenib as first‐line treatment.^32^, ^33^ Fifth, physicians decided to use second‐line and subsequent MTAs on demand. Thus, it will be necessary to perform a prospective study to determine the efficacy of sequential therapy and a comparison of the therapeutic effects of SORA and ICI treatment in patients with NASH or NAFLD HCC.

In conclusion, we demonstrated that after IPW, sequential therapy with SORA as the first‐line treatment prolongs survival for unresectable HCC patients. Moreover, sequential therapy with SORA as the first‐line treatment might be effective for patients regardless of HCC etiology.

## CONFLICT OF INTEREST

Atsushi Hiraoka received honoraria (lecture fees) from Bayer, Eisai, Eli Lilly, and Otsuka Pharmaceutical. Takumi Kawaguchi received honoraria (lecture fees) from Mitsubishi Tanabe Pharma Corporation and Otsuka Pharmaceutical Co., Ltd. The other authors declare no conflicts of interest.

## ETHICS APPROVAL STATEMENT

The study approval of the Ethical Committee of Kurume University School of Medicine (approval code: 21074) following the Helsinki Declaration.

## Supporting information

Figure S1

Figure S2

## Data Availability

Data are included in the manuscript or Supplementary Materials.
