# Supplementary figures and images for: First‐line sorafenib sequential therapy and liver disease etiology for unresectable hepatocellular carcinoma using inverse probability weighting: A multicenter retrospective study
Source: Cancer Med. 2021 Oct 24;10(23):8530–41. doi: 10.1002/cam4.4367 (PMC8633265; doi:10.1002/cam4.4367)

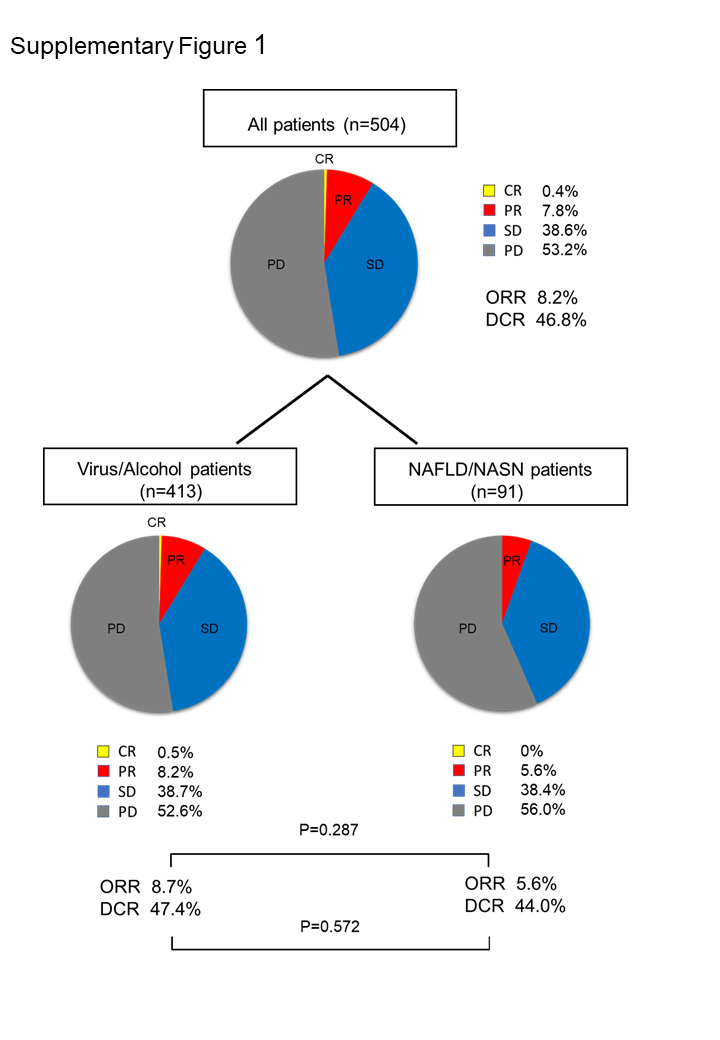

Supplement: Supplementary file 1 — Figure S1 [file CAM4-10-8530-s001.TIF]

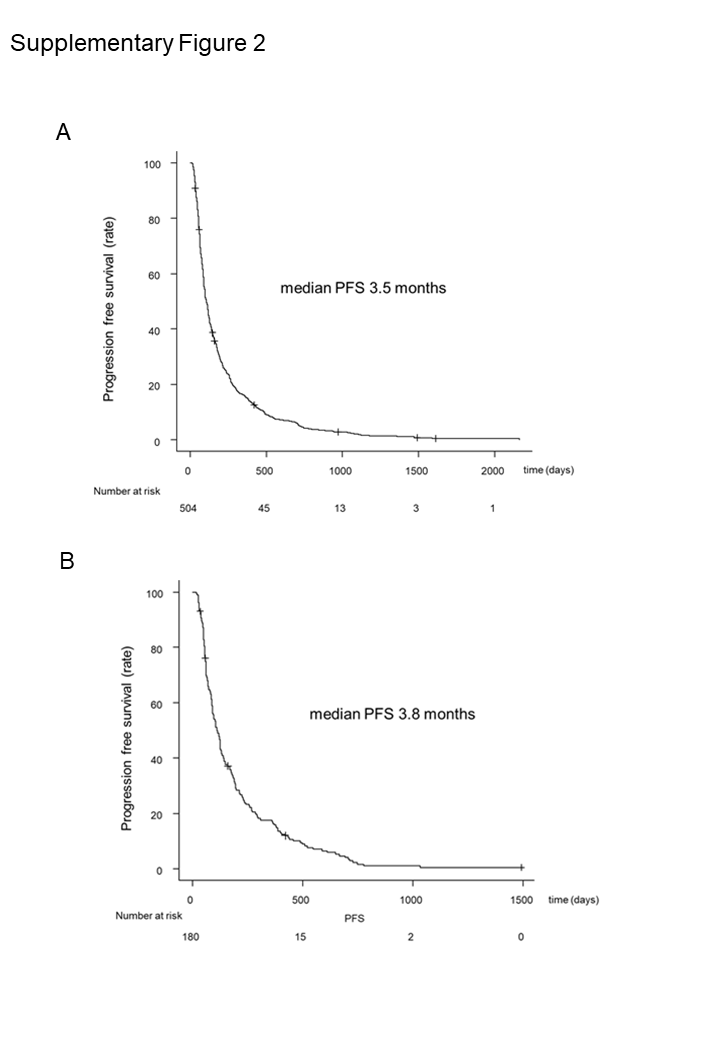

Supplement: Supplementary file 2 — Figure S2 [file CAM4-10-8530-s002.TIF]
